# Growth Patterns in MPS IVA and MPS IIIA: A Longitudinal Single-Center Study

**DOI:** 10.3390/jcm15114178

**Published:** 2026-05-28

**Authors:** Lior Carmon, Majd Nassar, Daphna Idan, Dar Leifman, David Shaki, Siham Elamour, Eli Hershkovitz, Neta Loewenthal, Alon Haim, Orna Staretz Chacham

**Affiliations:** 1 Pediatrics Endocrinology Unit, Saban Children’s Hospital, Soroka University Medical Center, Beer-Sheva 8410501, Israel; majdna@clalit.org.il (M.N.); davidsha@clalit.org.il (D.S.); elih@clalit.org.il (E.H.); netali@clalit.org.il (N.L.); alonhaim@clalit.org.il (A.H.); 2 Faculty of Health Sciences, Ben-Gurion University of the Negev, Beer-Sheva 8410501, Israel; daphnaid@post.bgu.ac.il (D.I.); darshosh@post.bgu.ac.il (D.L.); sihame@clalit.org.il (S.E.); ornasc@clalit.org.il (O.S.C.); 3 Pediatric Ambulatory Center, Saban Children’s Hospital, Soroka University Medical Center, Beer-Sheva 84101, Israel; 4 Rare Diseases Center, Saban Children’s Hospital, Soroka University Medical Center, Beer-Sheva 84101, Israel

**Keywords:** mucopolysaccharidoses (MPS), growth impairment, enzyme replacement therapy (ERT), lysosomal storage disorders, skeletal dysplasia

## Abstract

**Background/Objectives**: Mucopolysaccharidoses (MPS) are lysosomal storage disorders characterized by impaired glycosaminoglycan degradation, leading to multisystem involvement and progressive growth impairment. Longitudinal growth data in MPS IVA and MPS IIIA, including the association of ERT with growth outcomes, remain limited. This study aimed to characterize growth trajectories in MPS IVA and MPS IIIA and to assess the association of ERT with Elosulfase alfa on growth outcomes in MPS IVA patients. **Methods**: We retrospectively analyzed growth data from 39 patients with MPS subtypes IIIA and IVA followed at a single center between 2004 and 2024. Height and weight standard deviation scores (SDS) were calculated relative to CDC growth references and modeled using linear mixed-effects models (LMM). In the MPS IVA subgroup, the effect of ERT with Elosulfase alfa was assessed using LMM and paired SDS comparisons. **Results**: Growth impairment was evident across both subtypes with distinct trajectories. MPS IIIA patients showed significant height decline after age six with progressive weight loss in later childhood. MPS IVA patients exhibited persistently severe short stature and a tendency toward overweight with advancing age. Among the 16 MPS IVA patients treated with Elosulfase alfa who were included in the analysis, height SDS declined significantly during treatment (−0.127 SDS/year [95% CI: −0.194, −0.061], *p* < 0.001), and the rate of decline was not significantly affected by age at ERT initiation (interaction *p* = 0.53). **Conclusions**: ERT with Elosulfase alfa did not prevent progressive height loss relative to population norms. The rate of height SDS decline was not significantly influenced by the timing of ERT initiation (interaction *p* = 0.53), and causal conclusions cannot be drawn from this observational data.

## 1. Introduction

Mucopolysaccharidoses (MPS) are a heterogeneous group of inherited lysosomal storage disorders. Impaired activity of lysosomal enzymes leads to incomplete degradation and accumulation of glycosaminoglycans (GAGs). The accumulation of GAGs leads to damage in various tissues and organs [1]. Symptoms typically begin in childhood or even infancy and may include coarse facies, neurological impairment (depending on the MPS type), corneal clouding, hearing loss, obstructive airway disease and obstructive sleep apnea, recurrent respiratory infections, cardiac disease (mostly involving heart valves), hepatosplenomegaly, recurrent hernias and growth impairment [2].

The mechanism of growth impairment involves GAG accumulation in bone and cartilage cells, leading to dysfunction of these tissues [2,3]. Specifically, GAG accumulation results in impaired endochondral ossification with dysfunction in primary and secondary ossification centers, disrupted cell cycle progression in growth plate chondrocytes (specifically G1 to S phase transition), impaired chondrocyte proliferation and hypertrophy, diminished growth factor signaling and altered GAG-growth factor interactions, and increased cell stress and apoptosis.

On physical examination, characteristic features of patients with MPS include coarse facial features, short stature, short hands, skeletal deformities, and joint stiffness [2,4]. Recently, a diagnostic approach based on anthropometric measures has been proposed [5].

Previous studies have examined the growth patterns of patients with MPS and demonstrated severe growth impairment, with different growth trajectories among the various MPS types [2,4,6,7,8]. Growth hormone therapy has been suggested as a potential adjunct to improve linear growth in selected MPS patients with concurrent growth hormone deficiency [1]. The most prominent, MPS type IVA (Morquio syndrome), is caused by N-acetylgalactosamine-6-sulfate sulfatase (GALNS) deficiency due to various mutations in the *GALNS* gene [9]. Previous studies have shown significant height impairment among affected patients [8,10,11]. Bone density has also been found to be reduced [12].

In the last two decades, enzyme replacement therapy (ERT) has been shown to improve clinical outcomes in patients with MPS. ERT is available for several MPS types: Laronidase (ALDURAZYME^®^) for MPS type I, Idursulfase (ELAPRASE^®^) for MPS type II, Elosulfase alfa (VIMIZIM^®^) for MPS type IVA, Galsulfase (NAGLAZYME^®^) for MPS type VI and Vestronidase alfa (MEPSEVII^®^) for MPS VII.

Elosulfase alfa has been shown to improve clinical measures in MPS IVA [13]. The effect of this therapy on height has not been demonstrated to be significant [14] and is not considered a primary clinical target [15,16]. On the other hand, there is supporting evidence of modest height improvement with Elosulfase alfa treatment [17,18,19].

Our center follows patients with MPS subtypes IVA and IIIA, providing an opportunity to characterize longitudinal growth trajectories in both subtypes. This study had two primary objectives: first, to analytically characterize longitudinal growth trajectories in MPS IVA and MPS IIIA; and second, to assess, in an exploratory manner, the association of ERT with Elosulfase alfa with growth outcomes in MPS IVA patients, including the potential influence of age at treatment initiation.

## 2. Methods

This was a retrospective study. Data were obtained from digital medical records. The study period was 1 January 2004 through 1 March 2024. Inclusion criteria comprised all patients with a molecular diagnosis of MPS subtypes IVA or IIIA and adequate follow-up. Exclusion criteria included patients with additional chronic disorders or medications that could potentially impair linear growth, or patients with insufficient growth data. Data collection included demographic variables (date of birth, gender, ethnicity), and growth parameters from birth to the last medical visit, including height, weight, body mass index (BMI), and standard deviation scores relative to Centers for Disease Control and Prevention (CDC) growth charts. For patients with MPS type IVA, additional data were collected, including laboratory measures (insulin-like growth factor 1 [IGF-1] and growth hormone [GH] stimulation test results) and imaging studies (bone age). The study was approved by the local Helsinki Committee (protocol code SOR-0052-24). The authors used an artificial intelligence–based language model solely for English language editing and improvement of readability.

### Statistical Analysis

Serial height and weight measurements were plotted against gender- and age-specific growth percentiles (3rd through 97th) derived from the CDC 2000 growth reference for ages 2–20 years. Percentile curves were computed from the published lambda-mu-sigma (LMS) parameters. For MPS IV patients, BMI (kg/m^2^) was additionally plotted against CDC BMI-for-age percentiles. Height and weight standard deviation scores (SDS) were calculated relative to the CDC reference using the LMS method. Age was expressed in years for all modeling and visualization.

Growth trajectories were modeled using linear mixed-effects models (LMMs) to account for the correlation of repeated measurements within individual patients. In the pooled analysis, height and weight were each modeled as a function of age and gender, with a patient-specific random intercept and, when supported by the data, a random slope for age. The intraclass correlation coefficient (ICC) was derived from the variance components to quantify between-patient variability.

To visualize population-level growth trends on the percentile charts, predicted trajectories were derived from LMMs incorporating natural cubic spline terms for age. This approach allows the fitted curve to capture the non-linear pattern of growth rather than imposing a straight line. To ensure reproducibility, model selection followed a pre-specified fallback hierarchy: a natural cubic spline for age with random intercept and random slope was attempted first, and progressively simpler specifications (spline + random intercept; quadratic + random intercept; linear + random intercept) were used only if the more complex model failed to converge or was singular. The final model used for each subgroup is reported in Supplementary Table S1.

In the MPS IVA subgroup, the effect of ERT with Elosulfase alfa on growth was evaluated using two complementary approaches. The first approach employed an LMM to evaluate height and weight SDS trajectories over the course of ERT, including the potential influence of age at ERT initiation on the rate of SDS change.

In the second approach, height and weight SDS were compared individually for each of the 17 patients who received Elosulfase alfa; one patient was excluded due to insufficient data, leaving 16 patients for this analysis. For each patient, the measurement closest to the ERT start date and the most recent available measurement were used. The change in SDS (ΔSDS) was calculated for each patient, where a positive value indicates growth moving toward or above the population mean, and a negative value indicates further deviation from the norm. The Wilcoxon signed-rank test (paired) was used to test whether the within-patient change in height and weight SDS between ERT initiation and the most recent follow-up differed significantly from zero.

All analyses were performed in R version 4.5.1. Mixed-effects models were fitted using the lme4 and lmerTest packages [20]. CDC reference data and SDS calculations were obtained from the childsds package; spline basis functions were generated with the splines package. 95% confidence intervals for fixed-effect estimates were computed using profile likelihood; for paired ΔSDS, the 95% CI was derived from the within-patient distribution. Effect sizes are reported alongside CIs and contextualized in clinically interpretable units (cm/year, kg/year, SDS units, and projected SDS change over the mean treatment duration). A two-sided *p* < 0.05 was considered statistically significant.

## 3. Results

The analysis included 39 patients across two MPS subtypes—MPS IIIA (16 patients, 93 observations) and MPS IVA (23 patients, 290 observations), all aged ≥2 years, comprising 383 observations in total. Ninety-five percent of the cohort were of Bedouin ethnicity, and 62% were male. Patients had a median of 6 measurements each (range 1–43), with a mean of 9.8 measurements per patient.

Regarding height measurements in the entire cohort, the ICC was 96.1%, indicating that most of the total variance in height was explained by between-patient differences and that repeated measurements within the same patient were highly correlated, justifying the use of LMM. The pooled ICC of 96.1% reflects the substantial between-patient heterogeneity expected when two MPS subtypes with different growth phenotypes and severities are analyzed jointly. Within-patient longitudinal change is captured by the fixed-effect age slopes, not by the ICC; subtype-specific ICCs and slopes are reported in Supplementary Table S1. Full fixed-effect estimates with 95% confidence intervals from the pooled-cohort models are reported in Supplementary Table S2.

### 3.1. MPS IVA

Twenty-three patients with MPS IVA were included in the study, of whom molecular genetic data were available for 22 (95.7%). All patients with available data harbored variants in the *GALNS* gene (NM_000512.5). Two variants were identified: c.415G>A (p.Gly139Ser) and c.1474G>A (p.Ala492Thr). Seventeen patients were homozygous for c.1474G>A (p.Ala492Thr), three were homozygous for c.415G>A (p.Gly139Ser), and two were compound heterozygotes carrying both variants. One patient had a confirmed molecular diagnosis; however, the specific variant details were unavailable for this analysis.

**Figure 1:**
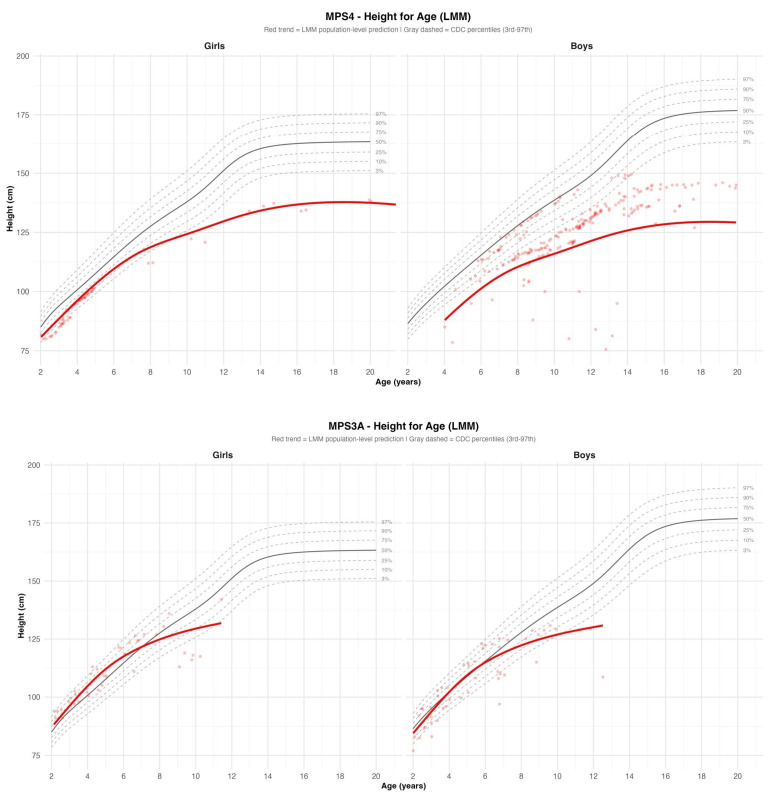
Height among MPS IIIA and MPS IVA patients.

**Figure 2:**
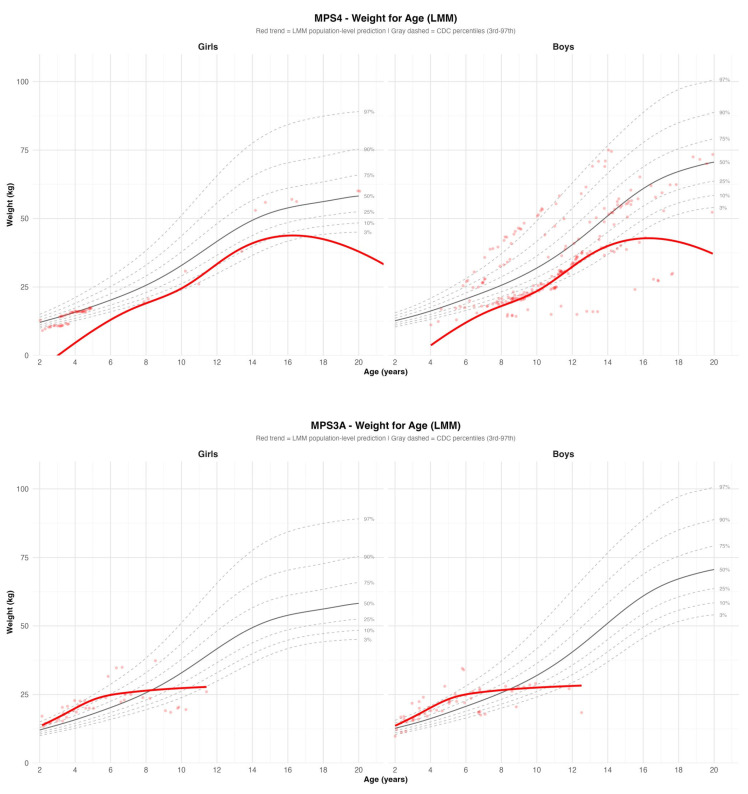
Weight among MPS IIIA and MPS IVA patients.

**Figure 3:**
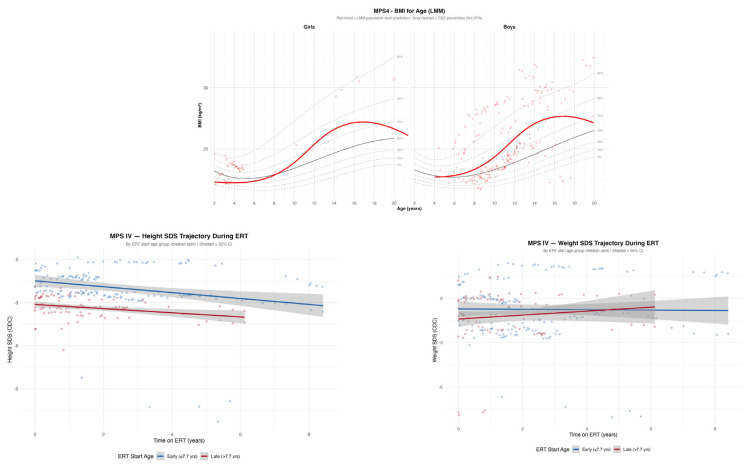
MPS IVA: BMI, height and weight SDS trajectory according to ERT initiating age.

Regarding growth parameters, all patients demonstrated a low mean height growth rate of 3.3 cm/year and a mean weight gain rate of 3.3 kg/year. Height SDS was low at baseline and declined progressively over time, while weight was markedly low in younger patients, followed by relative catch-up gain with age. No significant sex differences were observed (Figure 1 and Figure 2). BMI demonstrated a tendency toward overweight until approximately 15 years of age, followed by a decline thereafter (Figure 3).

Of the 23 patients in the MPS IVA subgroup, 17 received ERT with Elosulfase alfa; one was excluded due to insufficient data. The remaining 16 patients had a mean treatment duration of three years and six months, with a total of 250 measurements (mean 15.6 per patient). During the treatment period, the mean observed height growth rate was 6.44 cm/year [95% CI: 5.94, 6.94]. Height SDS declined at a rate of −0.127 SDS/year [95% CI: −0.194, −0.061] (*p* < 0.001), indicating progressive height loss relative to age- and sex-based norms, and this rate of decline was not significantly influenced by age at ERT initiation (interaction *p* = 0.53) (Figure 3; full model Supplementary Table S3). This trajectory-level finding was corroborated by the paired SDS comparison, which demonstrated a statistically significant within-patient decline in height SDS between ERT initiation and the most recent follow-up (mean ΔSDS −0.71 [95% CI: −1.11, −0.31], *p* = 0.0026, Table 1). Weight analysis revealed a similar pattern: weight SDS remained stable over time, with no significant change rate and no significant effect of ERT start age (−0.049 SDS/year [95% CI: −0.127, +0.029] interaction *p* = 0.52); the paired comparison similarly showed no significant change in weight SDS (mean ΔSDS −0.05 [95% CI: −0.44, +0.33], *p* = 0.8202, Table 1) (Figure 3).

Evaluation for GH deficiency was performed in four patients and was ruled out in all of them. IGF-1 levels were within the normal range in 11 of 14 patients (Table 1).

### 3.2. MPS IIIA

Sixteen patients with MPS IIIA were included in the study, with molecular genetic data available for all. All patients harbored variants in the *SGSH* gene (NM_000199.5). Fifteen of sixteen patients were homozygous for the c.267C>A variant. One patient was a compound heterozygote, carrying NM_000199.5:c.812C>T (p.Thr271Met) and NM_000199.5:c.1127dupT (p.Met376Ilefs*126). Regarding growth parameters, using the LMM, patients with MPS IIIA showed apparently normal height growth (~4.2 cm/year) and modest weight gain (~1.1 kg/year) during the follow-up period. No significant gender differences were observed for either parameter. However, when examining growth charts, height velocity declined around the age of 6 years in both boys and girls, and the height trend line dropped below the 3rd percentile. Weight was at high percentiles (around the 90th percentile) until approximately 6 years of age and subsequently declined in both genders to around the 10th percentile (Figure 1 and Figure 2).

## 4. Discussion

The following discussion addresses the two pre-specified objectives of this study: first, to analytically characterize longitudinal growth trajectories in MPS IVA and MPS IIIA, and second, to assess the effect of Elosulfase alfa on growth in MPS IVA.

### 4.1. MPS IVA

Morquio A syndrome is an autosomal recessive disorder caused by deficiency of the GALNS enzyme, resulting in accumulation of chondroitin-6-sulfate (C6S) and keratan sulfate (KS), and leading to multisystem involvement, particularly skeletal disease [10]. The predominance of two *GALNS* variants—c.1474G>A (p.Ala492Thr) and c.415G>A (p.Gly139Ser)—across the MPS IVA cohort is consistent with founder effects in this population. To our knowledge, the phenotypic consequences of these specific variants have not been systematically characterized in the published literature, and genotype–phenotype correlations for these variants therefore remain unknown. Regarding growth outcomes, patients in our cohort presented with low height and continued with persistent severe height impairment, consistent with previous studies [8,10,11] (Figure 1). With respect to weight, a previous study [8] reported slightly low average weight, though 6.8% of males and 5.4% of females were overweight, and the average BMI was higher than in the general population. In our cohort, patients began with low weight that increased over time (Figure 2). Relative weight to height (BMI) suggested a tendency toward overweight (Figure 3), similarly to the findings of the aforementioned study [8]; however, this trend was observed only up to age 15 years in our cohort. The weight gain seen in our cohort and in previous studies likely reflects the underlying pathophysiology: skeletal dysplasia leads to short stature with relatively preserved body mass and reduced mobility, predisposing to obesity. Obesity is a recognized concern in Morquio A syndrome and should be actively prevented [21]. Given the well-documented musculoskeletal burden in MPS IVA, prevention of excessive weight gain remains an important management consideration in this population, as additional load on already compromised joints may exacerbate functional decline. After age 15, however, weight and BMI declined relatively, possibly reflecting advanced disease, general deterioration, and impaired nutritional intake (Figure 2 and Figure 3).

Against this background of progressive skeletal and metabolic burden, the role of ERT in modifying growth trajectories warrants consideration. The literature presents somewhat mixed findings on its effect on growth. Doherty et al. reported no significant effect on height [14], while others have suggested the possibility of modest growth improvement with very early ERT initiation [17,18,19]. Overall, height SDS declined significantly relative to normative growth standards during ERT in our cohort, a result consistently demonstrated by both statistical approaches employed (Figure 3 and Table 1), likely reflecting the limited efficacy of ERT on cartilage, bone, and joint tissue due to poor drug penetration into these structures and their restricted blood supply [22]. Furthermore, the rate of height SDS decline was not significantly influenced by the timing of ERT initiation (interaction *p* = 0.53), indicating that earlier treatment initiation did not attenuate the progressive loss of height relative to population norms. It should be noted that, in the absence of an untreated control group, the observed decline in height SDS cannot be further characterized: it is not possible to determine whether it represents treatment failure, partial attenuation of a steeper natural decline, disease stabilization, or simply the expected untreated trajectory of MPS IVA. The study is therefore hypothesis-generating only with respect to ERT effects on growth. With respect to weight, SDS at the end of the treatment period was comparable to baseline values (Table 1 and Figure 3), suggesting that ERT does not substantially alter weight trajectory relative to population norms.

Exclusion of GH deficiency and normal IGF-1 levels among 11 of 14 patients (Table 1) support normal GH axis function in this population and are consistent with the assumption that short stature in these patients is primarily attributable to skeletal abnormality [2].

In three of six patients (the younger ones), bone age was mildly delayed relative to chronological age (Table 1), consistent with the abnormal endochondral ossification and growth plate maturation known to occur in this disease [2].

### 4.2. MPS IIIA

MPS IIIA (Sanfilippo syndrome) is an autosomal recessive disorder in which the accumulated GAG is heparan sulfate [2], resulting from deficient activity of the enzyme sulfamidase. It is characterized by neurodegeneration and physical features, such as joint stiffness and coarse facial features. The near-uniform presence of the *SGSH* c.267C>A variant across the MPS IIIA cohort is consistent with a founder effect in the Bedouin population. To our knowledge, the phenotypic consequences of this specific variant have not been systematically characterized in the published literature, and genotype–phenotype correlations therefore remain unknown. Previous studies have demonstrated growth impairment after the age of six years [2,23]. In our cohort, height declined significantly around the age of six years (Figure 1), consistent with previous reports [23]. In contrast, although a prior study [23] reported relatively high weight across all ages, weight in our cohort was initially elevated but declined around the age of six years (Figure 2), consistent with a report describing cachexia in these patients [24]. A mouse model study suggested that increased brown adipose tissue activity may contribute to the negative energy balance in advanced stages of the disease [25]. An additional explanation may relate to the disease pathophysiology, as MPS III involves progressive neurodegeneration that may impair feeding ability and nutritional status over time with disease deterioration and especially without the use of gastrostomy for nutrition.

### 4.3. Strengths and Limitations

Strengths of this study include a relatively large cohort including MPS IVA and MPS IIIA with sufficient longitudinal data for statistical modeling, a high number of longitudinal measurements, long-term follow-up, and the use of linear mixed-effects modeling to account for repeated measures. The availability of GH stimulation test and IGF-1 data in MPS IVA patients, as well as a dedicated analysis of Elosulfase alfa effects in this subgroup, represent further methodological strengths.

Limitations include the retrospective design and the presence of missing data. The absence of a non-treated control group limits causal inference; in our cohort, the vast majority of MPS IVA patients received ERT, leaving no untreated comparison group available. Consequently, it cannot be determined whether the observed SDS decline represents treatment failure, partial attenuation of decline, disease stabilization, or the expected natural history of MPS IVA. Comparisons based on age at ERT initiation are therefore subject to confounding, particularly by age and baseline disease severity. Key potential confounders—including disease severity, baseline skeletal involvement, and functional status—were not systematically available across the cohort and could not be incorporated into the analysis; their omission may limit the internal validity and mechanistic interpretation of the findings. The cohort was predominantly of Bedouin ethnicity (93%), which may limit generalizability to other populations and, in the absence of Bedouin-specific growth norms, may introduce additional bias in SDS interpretation when using CDC references derived from a non-Bedouin population. SDS calculations were additionally limited by the use of a healthy, unaffected reference population: in a disease characterized by severe skeletal dysplasia, declining SDS may partly reflect disease-specific dysplastic progression rather than treatment failure, and comparisons against unaffected normative data may overstate the apparent degree of deterioration. Disease-specific growth references for MPS IVA exist but have been derived from smaller and less representative cohorts; their use would limit comparability across studies. These considerations should be taken into account when interpreting the SDS-based findings reported here. Finally, CDC growth references are limited to ages 2–20 years; measurements obtained beyond this age range lacked calculable SDS values and were excluded from SDS-based analyses, introducing some degree of truncation bias for the small number of patients followed into adulthood.

## 5. Conclusions

This study analytically characterizes longitudinal growth trajectories in MPS IVA and MPS IIIA. ERT with Elosulfase alfa did not prevent progressive height loss relative to population norms. The rate of height SDS decline was not significantly influenced by the timing of ERT initiation (interaction *p* = 0.53), and these findings do not support a meaningful attenuation of growth impairment with earlier ERT initiation. Causal conclusions cannot be drawn from this observational data. Weight management warrants attention, as the tendency toward overweight in MPS IVA patients may exacerbate musculoskeletal burden and accelerate functional decline. Normal GH axis function in the majority of MPS IVA patients further supports skeletal dysplasia, rather than hormonal insufficiency, as the primary driver of short stature in this population. Taken together, these findings reinforce the importance of early diagnosis and long-term longitudinal growth monitoring in MPS IVA and MPS IIIA. Such monitoring may inform clinical management and contribute to a better characterization of disease progression and treatment response.

## Figures and Tables

**Table 1:** MPS IVA height and weight SDS before and after ERT treatment, laboratory and radiological data. Patients in the table—only treated or with available data.

Patient	Gender	IGF-1 (ng/mL)	Peak GH * (ng/mL)	Chronological Age/Bone Age (y)	Age at ERT Start	Height SDS at Start **	Weight SDS at Start **	Age at ERT End	Height SDS at ERT End **	Weight SDS at ERT End **	ERT Duration	Height SDS Change	Weight SDS Change
1	M	30.9 (40–255)	19.6		6 y 5 m	−1.88	−1.53	8 y 11 m	−2.77	−2.43	2 y 6 m	−0.89	−0.9
2	M	210 (143–506)	10.6	5.9/4–6	11 y 4 m	−2.73	−0.18	14 y 6 m	−3.3	0.24	3 y 2 m	−0.57	0.42
3	M				13 y 9 m	−3.55	−0.12	18 y 3 m	−4.39	0.24	4 y 6 m	−0.84	0.36
4	M	336 (191–496)			8 y 4 m	−3.35	−2.59	12 y 11 m	−3.58	−1.93	4 y 7 m	−0.23	0.66
5	M	31.2 (49.4–187.2)			7 y 5 m	−8.22	−6.69	9 y 2 m	−9.87	−8.01	1 y 9 m	−1.65	−1.32
6	F				3 y	−2.26	−0.06	4 y 6 m	−1.2	−0.22	1 y 6 m	1.06	−0.16
7	F	12.7 (15.6–65)	11.9		1 y 9 m	−1.85	−3.17	3 y 3 m	−2.27	−2.47	1 y 6 m	−0.42	0.7
8	M	225 (177–507)			5 y 9 m	−0.27	1.47	14 y 2 m	−1.9	1.69	8 y 5 m	−1.63	0.22
9	M	110 (68–310)		9/5–7	11 y 2 m	−2.57	−1.23	13 y 6 m	−2.78	−0.72	2 y 4 m	−0.21	0.51
10	M	204 (173–414)		16/17	7 y 8 m	−2.34	−2.01	16 y 5 m	−3.63	−2.41	8 y 9 m	−1.29	−0.4
11 ^#^	F	166 (99–289)			15 y 7 m	−3.63	−1.26	23 y 10 m	NA	NA	8 y 3 m	NA	NA
12	M	101 (40–255)		8/8	6 y 11 m	−0.75	0.93	8 y 9 m	−0.91	1.38	1 y 10 m	−0.16	0.45
13	M	190 (19–487)		17/19	15 y 1 m	−3.11	−0.67	19 y 10 m	−4.56	−2.13	4 y 9 m	−1.45	−1.46
14	F	59.8 (56.9–277)	10.4	7.8/5–6.10									
15	F				15 y 3 m	−3.76	0.44	16 y 3 m	−4.38	0.18	1 y	−0.62	−0.26
16	M				11 y 6 m	−1.89	1.21	12 y6 m	−2.28	1.47	1 y	−0.39	0.26
17	M	205 (173–414)			16 y 10 m	−4.87	−7.74	17 y 7 m	−6.3	−7.59	9 m	−1.43	0.15
Mean					9 y 10 m	−2.89	−1.46	13 y 5 m	−3.61	−1.51	3 y 6 m	−0.71	−0.05
95% CI												−1.11, −0.31	−0.44, +0.33
*p*-value												0.0026	0.8202

SDS calculated using CDC 2000 reference. * s/p stimulation test. ** Measurement closest to ERT start or end date. Mean calculated from patients with complete data only. *p*-values from paired Wilcoxon signed-rank test (testing whether delta SDS differs from zero). ^#^ Patient 11: last visit age 24 years, exceeds CDC reference range (2–20 years), SDS not calculable. Abbreviations: ERT, enzyme replacement therapy; SDS, standard deviation score; y, years; m, months; IGF-1, insulin growth factor 1; GH, growth hormone.

## Data Availability

The data presented in this study are available on request from the corresponding author due to privacy and ethical restrictions.

## Supplementary Materials

The following supporting information can be downloaded at https://www.mdpi.com/article/10.3390/jcm15114178/s1, Table S1: Per-MPS-subtype linear mixed-effects models for height and weight: model selection, sample size, and intraclass correlation coefficients; Table S2: Fixed-effect estimates from the pooled-cohort linear mixed-effects models for height and weight (all five MPS subtypes combined; n = 44 patients, 421 observations); Table S3: Linear mixed-effects model fixed-effect estimates for the MPS IVA on-ERT subgroup, modeling absolute and SDS-standardized height and weight as functions of time on ERT, age at ERT initiation, their interaction, and gender.

## Abbreviations

The following abbreviations are used in this manuscript:

BMI	body mass index
C6S	chondroitin-6-sulfate
CDC	Centers for Disease Control and Prevention
ERT	enzyme replacement therapy
GALNS	N-acetylgalactosamine-6-sulfate sulfatase
GAGs	glycosaminoglycans
GH	growth hormone
ICC	intraclass correlation coefficient
IGF-1	insulin-like growth factor 1
KS	keratan sulfate
LMM	linear mixed-effects model
LMS	lambda-mu-sigma
MPS	mucopolysaccharidoses
MPS3A	mucopolysaccharidosis type IIIA (Sanfilippo A syndrome)
MPS4	mucopolysaccharidosis type IVA (Morquio syndrome)
SDS	standard deviation score
ΔSDS	change in standard deviation score
