# Supplementary material for: Growth Patterns in MPS IVA and MPS IIIA: A Longitudinal Single-Center Study
Source: J Clin Med. 2026 May 28;15(11):4178. doi: 10.3390/jcm15114178 (PMC13258356; doi:10.3390/jcm15114178)
Supplement: Supplementary file 1 [file jcm-15-04178-s001.zip › jcm-4298480-supplementary.pdf]

Supplementary Materials

Supplementary Table S1. Per-MPS-subtype linear mixed-effects models for height and weight: model selection, sample size, and intraclass correlation coefficients.

MPS subtype	Outcome	N patients	N observations	Final model retained	ICC
**MPS IIIA**	Height (cm)	16	93	L1: spline (df = 4) + RI + RS	0.563
**MPS IIIA**	Weight (kg)	16	93	L1: spline (df = 4) + RI + RS	0.664
**MPS IVA**	Height (cm)	23	290	L1: spline (df = 4) + RI + RS	0.937
**MPS IVA**	Weight (kg)	23	290	L1: spline (df = 4) + RI + RS	0.922

Model selection followed a pre-specified fallback hierarchy: L1 - natural cubic spline for age + random intercept (RI) + random slope (RS); L2 - spline + RI; L3 - quadratic + RI; L4 - linear + RI. The most flexible model (L1) was retained for all four subgroup × outcome combinations; no fallback was required. ICC = intraclass correlation coefficient. RI = random intercept; RS = random slope.

Supplementary Table S2. Fixed-effect estimates from the pooled-cohort linear mixed-effects models for height and weight (MPS IIIA and MPS IVA combined; n = 39 patients, 383 observations).

Outcome	Fixed effect	Estimate	95% CI
**Height (cm)**	Intercept	89.72	82.43, 97.39
	Age (per year)	4.07	3.22, 4.93
	Gender (male vs female)	−6.05	−13.68, 1.47
**Weight (kg)**	Intercept	10.87	6.66, 15.01
	Age (per year)	2.21	1.64, 2.76
	Gender (male vs female)	−5.13	−9.48, −0.86

Models: outcome ~ age + gender + (1 + age | patient_number). 95% confidence intervals were computed using profile likelihood. CI = confidence interval.

Supplementary Table S3. Linear mixed-effects model fixed-effect estimates for the MPS IVA on-ERT subgroup, modelling absolute and SDS-standardised height and weight as functions of time on ERT, age at ERT initiation, their interaction, and gender.

Outcome	Fixed effect	Estimate	95% CI
**Absolute height (cm)**	Time on ERT (per year)	6.44	5.94, 6.94
	Age at ERT start (per year)	3.69	2.22, 5.16
	Time on ERT × age at start	−0.346	−0.402, −0.290
	Gender (male)	7.28	−7.91, 22.46
**Height SDS**	Time on ERT (per year)	−0.127	−0.194, −0.061
	Age at ERT start (per year)	−0.157	−0.386, 0.072

Outcome	Fixed effect	Estimate	95% CI
	Time on ERT × age at start	−0.0024	−0.0101, 0.0053
	Gender (male)	−0.18	−2.55, 2.18
**Absolute weight (kg)**	Time on ERT (per year)	6.03	5.34, 6.73
	Age at ERT start (per year)	3.14	1.73, 4.56
	Time on ERT × age at start	−0.284	−0.361, −0.206
	Gender (male)	−1.24	−15.81, 13.34
**Weight SDS**	Time on ERT (per year)	−0.049	−0.127, 0.029
	Age at ERT start (per year)	−0.032	−0.345, 0.282
	Time on ERT × age at start	0.0029	−0.0060, 0.0119
	Gender (male)	−0.93	−4.18, 2.31

Model form: outcome ~ time_on_ert × ert_start_age + gender + (1 | patient_number). Fitted on on-ERT measurements only (16 patients, 250 observations after exclusion of one patient with insufficient data). 95% confidence intervals computed using profile likelihood. SDS rows are shaded for visual separation. SDS = standard deviation score (CDC 2000 reference); ERT = enzyme replacement therapy.
